# Conversations at the crossroads of the Human RNome Project: a collaborative reflection by the RNome Early Career Researchers

**DOI:** 10.1080/15476286.2026.2613884

**Published:** 2026-01-07

**Authors:** Bennett Henzeler, Rebekah Penrice-Randal, Rami Bechara, Özge Simsir, Shanice Jessica Hermon, RNome Early Career Researcher group#

**Affiliations:** a Department of Chemistry, Institute for Chemical Epigenetics, Ludwig-Maximilians University, Munich, Germany; b Centre for Proteome Research, Department of Biochemistry, Cell & Systems Biology, Institute of Systems, Molecular & Integrative Biology, University of Liverpool, Liverpool, UK; c Inserm, CEA, Center for Immunology of Viral, Auto-Immune, Hematological and Bacterial Diseases (IMVA-HB/IDMIT), Université Paris-Saclay, Le Kremlin-Bicêtre, France; d Max Planck Institute of Molecular Physiology, Dortmund, Germany; e Department of Chemistry and Chemical Biology, Technical University Dortmund, Dortmund, Germany

**Keywords:** Human RNome Project, early career researcher, RNA biology, epitranscriptomics, collaboration, consortium

## Inception of the Human RNome Early Career Researcher (ECR) initiative: next decade of RNA science

**Figure 1.**
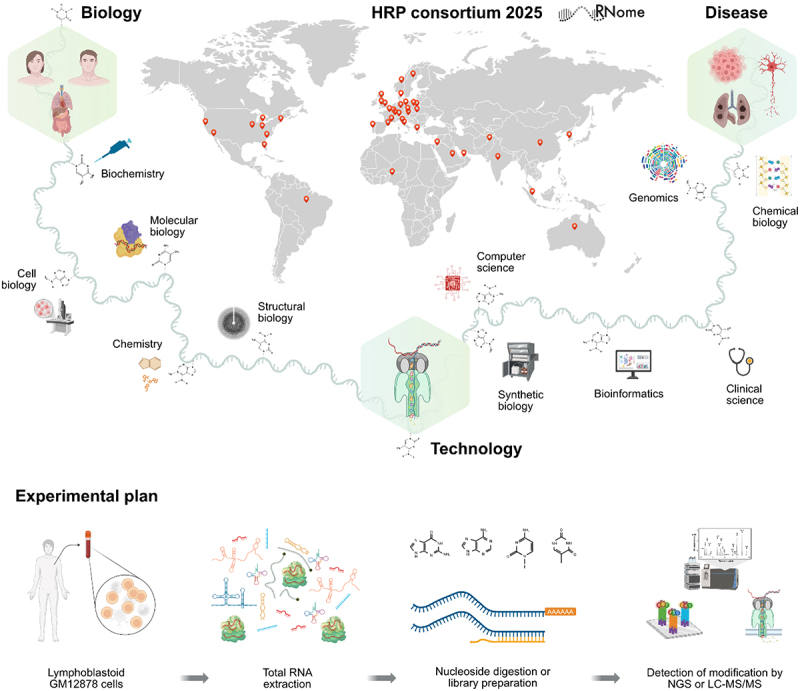
Overview of the Human RNome project and multidisciplinary scope.

As early-career researchers, we often balance the excitement of discovery with the uncertainty of shaping our scientific paths. Meetings that bring us together across disciplines are more than opportunities to share data; they are spaces to spark ideas, build networks, and gain perspectives from others who are navigating similar challenges. The recent 2025 Human RNome Project (HRP) Consortium gathering, held from 4–8 August at Goethe University Frankfurt, Germany, was exactly that. It was a space where established leaders and newcomers in RNA biology could connect, exchange insights, and envision experiments that might shape the next decade. HRP is a global effort to reveal the complete landscape of human RNA biology (Figure 1). The consortium gathering resulted in three defining outcomes. First, it built strong momentum towards sequencing RNA with all its chemical modifications, a key step towards mapping the complete human RNome. Second, it established a harmonized protocol for ribonucleoside identification and quantitation, ensuring consistency and comparability across laboratories. Third, it launched a collaborative effort to generate benchmark human RNome sequences. Alongside these scientific milestones, the meeting also marked the formation of the RNome ECR group. The origins of this initiative can be traced to an engaging discussion during one of the breakout sessions, an extension of the inspiring teaching platform fostered by the founding members of the RNome consortium [1].

This figure presents the experimental workflow and multidisciplinary scope of the RNome project, conducted under the HRP Consortium. The process initiates with total RNA extraction from lymphoblastoid GM12878 cells, followed by either nucleoside digestion or library preparation. RNA modifications are subsequently identified using Next-Generation Sequencing (NGS) or Liquid Chromatography–Tandem Mass Spectrometry (LC-MS/MS). The illustration also reflects the project’s integration across a wide range of scientific disciplines including genomics, chemical biology, computer science, structural biology, and clinical research underscoring a systems-level approach to the study of RNA modifications.

This piece offers perspectives from the ECR group of the inaugural workshop. Following the formation of the RNome ECR group, our first steering meeting set the direction for this emerging initiative. The discussion’s outcome is captured in a conversational profile, reflecting the HRP through the eyes of its next generation of researchers. When the Human Genome Project (HGP) concluded, it was rightly celebrated as one of biology’s greatest achievements, providing a blueprint for understanding human life. Yet even as we recognized that milestone, a question lingered in our RNA-focused minds. *‘What happens to the RNA? It serves as the bridge between DNA and protein, translating static code into dynamic function, yet its complexity, particularly the impact of its chemical modifications, remains largely unexplored. Throughout my scientific journey, I have been fascinated by these regulatory layers, from studying histone deacetylation during viral infections to characterising modified small RNAs. When I first heard about the HRP workshop in Frankfurt, I registered out of curiosity, expecting little more than networking opportunities and a few new ideas. What I found instead was transformative’* - B. Henzeler. *‘It was more than a meeting; it was the beginning of a collective movement to connect researchers across academia and industry around a shared goal of understanding RNA modifications in all their complexity. The breadth of interest from more than a hundred participants showed the true scale of enthusiasm in this field’* - R. Penrice-Randal. In the following reflections, several of us share our personal takeaways from the week, each from a different background, yet united by the belief that these conversations matter as much as the science itself. HRP is a long-term endeavour aspiring to achieve an impact akin to the HGP.

## HRP consortium: mission to sequence RNA with all the modifications

Since the discovery of 5-methylcytidine and pseudouridine in the 1960s, more than 180 RNA modifications have been identified across organisms, including over 50 distinct marks in humans [2,3]. Presented in all RNA types, these chemical modifications influence structure, stability, and RNA–protein interactions, forming a molecular bridge between transcription and translation [4]. RNA biology has thus entered a new era, recognizing that these modifications regulate RNA processing, localization and function. The HRP marked a defining moment for the RNA research community, bringing together scientists from across disciplines to advance the shared mission of understanding the human RNome. By providing the first comprehensive, modification-resolved view of the human RNome, the HRP will lay the foundation for new discoveries, drive innovation across biology and biotechnology, and open new frontiers in therapeutic development. The HRP’s story began with the commentary, which articulated a fundamental challenge in molecular biology [5]. That call for technologies capable of sequencing full-length RNAs in their native, modified form marked the inception of the HRP. ‘*While complete DNA sequences exist for most organisms, their full RNA sequences-complete with chemical modifications-remain unknown. I began speaking with researchers pioneering ways to sequence RNA with its modifications intact, and suddenly, ideas that had once seemed hypothetical began to feel possible. Those conversations changed my perspective entirely*.’ - R. Bechara.

HRP draws inspiration from a landmark National Academies of Sciences, Engineering, and Medicine (NASEM) report; one modelled after the document that launched the genome era in 2003 [6]. RNome is far more complex than the genome: RNA molecules undergo frequent chemical modifications, meaning there can never be a single, fixed reference sequence. Each RNA must be characterized not only by its nucleotide sequence, but also by the identity and position of its modifications [7]. HRP aims to catalogue RNA modifications across model organisms and human cell lines, supported by dedicated ‘RNA core centres’. Complementary goals include creating standardized synthetic RNAs, improving mass spectrometry and chromatography methods, and building centralized international databases. On 8–10 January 2024, the HRP Consortium convened its inaugural meeting in Rhode Island, marking the official launch of the initiative. Discussions centred on establishing standards for technology development, reference cell lines, data formats and databases [8]. The next instalment of this meeting was this year’s HRP Consortium gathering, a workshop dedicated to identifying and quantifying RNA chemical modifications through harmonized analyses across three mass spectrometry platforms. What made this gathering particularly impactful was its dual emphasis on technical innovation and community coordination. The workshop was thoughtfully structured, incorporating breakout sessions alongside harmonization and training modules to foster both learning and collaboration. ‘*The consensus was clear: a complete understanding of RNA biology will require technologies that can integrate chemical context with sequence information. This realization has since become a central pillar of the Human RNome Project’s vision’*. - Ö. Simsir

It was not simply a meeting to exchange data; it was an effort to align methodologies, harmonize analytical standards, and lay the foundation for the next phase of RNA biology. One of the most important outcomes of the workshop was the collective drive to accelerate sequencing efforts that can capture RNA with all of its chemical modifications intact. This goal represents the next frontier in transcriptomics. Traditional sequencing approaches, while powerful, often overlook the chemical marks that regulate stability, translation, and function [9,10]. Recognizing this gap, researchers at the workshop discussed strategies to build sequencing platforms capable of resolving these modifications directly. Another key achievement was the establishment of a harmonized protocol for ribonucleoside identification and quantitation [11]. Standardizing these procedures across laboratories is essential for ensuring that the results are comparable and reproducible [11]. Participants worked collaboratively to refine workflows for mass spectrometry-based detection of RNA modifications, addressing technical challenges such as ionization variability and chromatographic consistency [12,13]. This commitment to harmonization reflects a broader shift within the field towards open science and shared infrastructure. The 2025 workshop also marked the launch of an ambitious collaborative effort to draft reference human RNome sequences. These benchmark datasets will serve as a foundation for future studies that explore RNA diversity, chemical modifications, and functional regulation across tissues and disease states. Participants emphasized that such a resource must be both inclusive and adaptable, accommodating emerging sequencing technologies and new analytical tools. The discussion underscored that the HRP is not a single experiment, but a sustained, evolving collaboration that will grow as the field matures. Beyond the technical outcomes, the 2025 HRP workshop fostered a sense of shared purpose that extended well beyond the meeting rooms. Conversations that began during experimental sessions continued over coffee and late into the evenings, evolving into concrete collaborations and new project proposals. ‘*If the Human Genome Project gave us a map of our genetic code, the Human RNome Project will provide us with a map of life’s dynamic regulation. It is a story not only of molecules but of movement, modification, and meaning. Many voices will write that story, each contributing a unique perspective. The ECR community represents the next generation of those voices-scientists committed to openness, collaboration, and curiosity. Together, we are not only continuing the work but redefining how it is done’*. - S. J. Hermon

## From one generation to the next: the torch of the human RNome

To ensure these themes were explored with both depth and perspective, the 2025 HRP consortium meeting unfolded through a series of dynamic breakout sessions designed to spark focused discussion and collective insight. Over five days, ten sessions were led by experts at various career stages, fostering a vibrant exchange of ideas. Dedicated note-takers captured key takeaways from each discussion, ensuring that the voices of participants will directly inform the next phase of HRP planning. One of the sessions was organized entirely by ECRs, chaired by R. Penrice-Randal and R. Bechara, the session-‘*Data Meets Lab: Bridging Experimentation and Analysis*’-focused on the infrastructural and collaborative challenges of managing mass spectrometry data and raw Oxford Nanopore (ONT) reads in RNA modification research, given the current absence of a suitable repository or database. ‘*On my very first day, the first person I spoke with was Rami, with whom I would later co-facilitate a session. Discovering that they, too, were an early-career researcher and relatively new to the world of RNA modification was both reassuring and motivating. It quieted some of my initial imposter feelings and gave me the confidence to engage more fully with the week ahead*.’ - R. Penrice-Randal. A survey conducted during the meeting highlighted ongoing friction between wet-lab and computational teams, often stemming from inconsistent sample labelling, incomplete metadata and ambiguous terminology.

This session planted the seed for many ECRs in the room to recognize that they represent the future strength of the HRP. Fast forward to the final two days of the consortium gathering, where simultaneous sessions were hosted by Fred Tyson and Vivian Cheung, and by Silvio Conticello and Peter Dedon, further shaping the collaborative direction of the HRP. The sessions invited participants to envision the state of RNome science in the year 2030. Discussions revolved around three guiding questions: What will we be able to do technologically? What will we know scientifically? And what will we have changed clinically and societally? From a technological standpoint, participants imagined single-molecule approaches capable of detecting all RNA isoforms and their modifications at base-level resolution, with precision and error rates comparable to DNA sequencing. Advancements in nanopore chemistries, innovative ligases and increasingly sensitive mass spectrometry platforms were highlighted as key areas for innovation. The foundational HRP workshop culminated in a unified action plan centred on a first collaborative project; to map RNA modifications using a shared stock of total RNA from ENCODE GM12878 B-cells [14] as a standardized reference sample. In parallel, the consortium also has plans to develop a shared resource hub on the HRP website to host standardized protocols, kit recommendations, and reagent sources. Finally, participants identified the need for dedicated project management to coordinate logistics and oversee the growing complexity of this international collaboration.

The meeting concluded with a clear plan for the next phase. One lab will share RNA samples with participating members, who will analyse them using their own established methods. The resulting datasets will be combined and compared, a pilot study to kick-start this global effort. Three teams were formed to focus on technology development, biological functions, and disease relevance. The goal is ambitious, perhaps even daunting, but the group’s energy and commitment makes it feel achievable.

## The afterburn of the 2025 HRP gathering: the human side of the human RNome

From the very first day, the vision was clear: to map the human epitranscriptome together. The discussions were intense but full of energy, fuelled by curiosity and mutual respect. The most impressive part was the diversity, not only of expertise but of perspective. Biologists, chemists, bioinformaticians, computational scientists, and technology developers all sitting together, debating methods, sharing ideas and building on each other’s thoughts. ‘*As one of the youngest postdocs, I was genuinely inspired by how warmly I was welcomed into the RNome Consortium. Being ambitious and proactive has always guided my career-and once again, it opened new doors, leading me to become a founding member of the RNome Early Career Researcher group. Senior researchers engaged me in conversation, sought my input and valued every contribution. That openness gave me both confidence and a true sense of belonging*’ - B. Henzeler. Leaving Frankfurt, it was evident that the Human RNome Project is defined as much by its people as by its science. Even as the youngest attendees, we felt welcomed, heard, and empowered to contribute, giving rise to the formation of the RNome Early Career Researcher group. *‘Seeing this consortium in action made it clear that when individuals come together with a shared purpose, the results can far exceed what any of us could do alone’*. - S. J. Hermon. Frankfurt showed us that RNA science thrives on collaboration, curiosity, and trust. Energized and inspired, we now carry that momentum forward, ready to shape the next decade of the Human RNome Project. Looking back, the 2025 Human RNome Project meeting in Frankfurt felt like the start of an extremely large group project, one that no one wants to drop out of. ‘*At some point between the coffee breaks and data discussions, I realized the Human RNome Project is less about RNA and more about the humans trying to make sense of it’. -* R. Bechara. The birth of the RNome ECR group captured that spirit perfectly. It reminded us that science moves faster (and is a lot more fun) when ECRs are not just observers but active drivers of the field. The next decade of RNA research will no doubt bring new tools and discoveries, but also new challenges, and more acronyms than anyone can keep track of. Still, the energy and openness we felt in Frankfurt gave real confidence that this community can handle it. We are not just decoding RNA; we are learning how to work together better and build a future for RNA science that stays driven by people, not just data.
